# APPL1 Is a Prognostic Biomarker and Correlated with Treg Cell Infiltration via Oxygen-Consuming Metabolism in Renal Clear Cell Carcinoma

**DOI:** 10.1155/2023/5885203

**Published:** 2023-02-17

**Authors:** Ming Yang, Chuhui Gong, Kangping Song, Ning Huang, Honghan Chen, Hui Gong, Yu Yang, Shujing Guo, Hengyi Xiao

**Affiliations:** ^1^The Lab of Aging Research, State Key Laboratory of Biotherapy, National Clinical Research Center for Geriatrics, West China Hospital, Sichuan University, Chengdu, China; ^2^Rehabilitation Medicine Center, West China Hospital, Sichuan University, Chengdu, China; ^3^Department of Health Management & Institute of Health Management, Sichuan Provincial People's Hospital, University of Electronic Science and Technology of China, Chengdu, China

## Abstract

Kidney renal clear cell carcinoma (KIRC) is one of the most hazardous tumors in the urinary system. The regulation of oxygen consumption in renal clear cell carcinoma is a consequence of adaptive reprogramming of oxidative metabolism in tumor cells. APPL1 is a signaling adaptor involved in cell survival, oxidative stress, inflammation, and energy metabolism. However, the correlation of APPL1 with regulatory T cell (Treg) infiltration and prognostic value in KIRC remain unclear. In this study, we comprehensively predicted the potential function and prognostic value of APPL1 in KIRC. For KIRC patients, relatively low expression of APPL1 was associated with high degree of metastasis, pathological stage, and shorter overall time or poor prognosis. Gene Ontology (GO) and Kyoto Encyclopedia of Genes and Genomes (KEGG) enrichment analyses suggested that low expression of APPL1 may be adapted to the malignant progression of tumors via affecting oxygen-consuming metabolism. In addition, the expression level of APPL1 was negatively correlated with Treg cell infiltration and chemotherapy sensitivity, which indicated that APPL1 may regulate the tumor immune infiltration and chemotherapy resistance by decrease oxygen-consuming metabolic process in KIRC. Therefore, APPL1 may become one of the important prognostic factors, and it may serve as a candidate prognostic biomarker in KIRC.

## 1. Introduction

Renal cell carcinoma (RCC) is a lethal cancer of the urinary system, which is second only to prostate and bladder cancer in mortality worldwide [1–3]. Kidney renal clear cell carcinoma (KIRC) is a predominant heterogeneous histological subtype (~70%) of kidney cancer and is one of the most malignant diseases [4, 5]. Tumor metastases occur in approximately 30% in diagnosed KIRC patients, and it has a 40% of recurrence rate after surgery [6, 7]. Currently, patients with KIRC could obtain a favorable prognosis after chemotherapy, radiotherapy, and especially immunotherapy and chemotherapy, but chemotherapy resistance is still a very challenging issue [8–10]. The poor prognosis of KIRC may be related to the reprogramming of energy metabolism in tumor cells, such as the downregulation of the oxygen-consuming tricarboxylic acid cycle (TAC) and damage caused by oxidative stress [11]. The immune system can inhibit tumor cell growth, while some T cells from tumor microenvironment can also promote tumor malignant progression via promoting immunosuppressive state [12]. Numerous studies have shown that CD4^+^ T cells, CD8^+^ T cells, etc. played dual roles in KIRC: functioning as antitumor or leading to a poor prognosis [12–14]. Therefore, there is an urgent need to clarify the immunophenotype of tumor-immune interactions and related therapeutic targets for improving the prognosis of KIRC therapy.

Adaptor protein containing a pH domain, a PTB domain, and a leucine zipper motif 1 (APPL1) is a mediator protein that associates with regulation of cellular energy metabolism, survival, oxidative stress, and inflammation regulation [15–17]. In addition, the APPL1/AMPK or APPL1/Nrf2 pathways promote the uptake and metabolism of glucose and fatty acids for anti-inflammatory and antioxidant responses in normal cells [18, 19]. Thus, expression level of APPL1 may be correlated with change of tumor microenvironment in KIRC. However, the mechanism and poor prognosis of APPL1 expression on Treg cell infiltration in KIRC remain unclear.

In this study, we integrated multiple bioinformatics methods based on database online to explore whether APPL1 affects tumor Treg infiltration and try to find its correlation with poor prognosis in KIRC patients. We found that the expression of APPL1 was significantly downregulated in KIRC tumor tissues compared with adjacent normal tissues. Besides, the expression level of APPL1 was correlated with survival rate and Treg infiltration and immune checkpoints in KIRC. Our further analysis revealed that APPL1 and related genes may affect the oxygen-consuming metabolic process, which contribute to chemotherapy resistance of KIRC patients. These results highlight that APPL1 may serve as a biomarker in predicting the prognosis of KIRC.

## 2. Materials and Methods

### 2.1. APPL1 Expression Analysis

APPL1 mRNA expression level data for 33 tumor types were obtained from the TIMER2.0 online database (http://timer.comp-genomics.org). The RNA-seq transcriptome data and related clinical information were obtained from The Cancer Genome Atlas (TCGA) database (https://portal.gdc.cancer.gov/). There are 539 KIRC samples and 72 adjacent normal samples included in this study. Statistical analyses were performed using the R software v4.0.3 (R Foundation for Statistical Computing, Vienna, Austria), ^∗^*p* < 0.05, ^∗∗^*p* < 0.01, and ^∗∗∗^*p* < 0.001.

The data on KIRC tumor protein expression levels were obtained from Clinical Proteomic Tumor Analysis Consortium (CPTAC). The difference of APPL1 mRNA and protein level for tumor tissue and adjacent normal tissue in KIRC was analyzed via UALCAN database online (http://Ualcan.path.uab.edu/analysis). We also investigate the various clinicopathological parameters for KIRC (gender, tumor stage, lymph node metastasis status, etc.) in UALCAN.

### 2.2. Cell Migration and Proliferation Assays

The culture conditions of 786O, 769P, and Caki-1 cells were DMEM-F12 (90%, Gibcol) + FBS (10%, Gibcol) + penicillin and streptomycin (1%, Biosharp). Scratch wound and Transwell assay were used in cell migration experiments. The scratch wound assay was photographed at 0 h and 24 h, respectively; Transwell was fixed with 4% paraformaldehyde and stained with crystal violet at 24 h. The proliferation assay of cells in each group was conducted by adding CCK8 (DOJINDO) to determine the OD450 absorption value from 1 to 5 days. All experiments were independently repeated three times.

### 2.3. Kaplan-Meier Plotter and ROC Analysis

RNA-sequencing expression (level 3) profiles and related clinical information for KIRC were from TCGA dataset (https://portal.gdc.com). Log-rank test was used to compare differences in overall survival (OS), progression-free interval (PFI), and disease specific survival (DSS) between these groups, and it compared the predictive accuracy of APPL1 mRNA by the time ROC (v 0.4). For the Kaplan-Meier curves, *p* values and hazard ratio (HR) with 95% confidence interval (CI) were generated by log-rank tests and univariate Cox proportional hazards regression.

### 2.4. Prognostic Model (Nomogram)

Univariate and multivariate cox regression analyses were performed to identify the proper terms to construct the nomogram. The forest plot was used to display the *p* value, HR, and 95% CI of each variable via R package (forest plot). The nomogram was constructed based on the results of multivariate cox proportional hazards analysis to predict the 1-, 3-, 5-year overall recurrence. The nomogram provided a graphical representation of the factors, which can be used to calculate the risk of recurrence for an individual patient by R package (rms).

### 2.5. Correlation Analysis

Immune correlation analysis of APPL1 expression and the two-gene correlation map in KIRC were conducted by the R software gg stats plot package. We used Spearman's correlation analysis to describe the correlation between quantitative variables without a normal distribution. All the analysis methods and R package were implemented by R version (^∗^*p* < 0.05, ^∗∗^*p* < 0.01, and ^∗∗∗^*p* < 0.001). TISIDB is a web portal for tumor and immune system interaction, which integrates multiple heterogeneous data types. Immune checkpoint analysis of APPL1 expression in KIRC was conducted through TISIDB database online (http://cis.hku.hk/TISIDB/index.php).

We used the R software GSVA package to analyze, choosing parameter as method = “ssgsea.” The correlation between genes and pathway scores was analyzed via Spearman's correlation. All the analysis methods and R packages were implemented by R version 4.0.3 (^∗^*p* < 0.05, ^∗∗^*p* < 0.01, and ^∗∗∗^*p* < 0.001).

### 2.6. Enrichment Analysis of Genes Correlated with APPL1

Metascape (https://metascape.org) is a tool that integrated the enrichment functions of GO and KEGG [20]. The genes positively correlated with APPL1 expression were analyzed, and we obtain the related biological processes and pathways. The *p* < 0.01 and an enrichment factor > 1.5 indicate significance of the results. GSEA enrichment analysis was conducted by software R (version 3.6.3) and ggplot2 package (version 3.3.3), and the threshold for significant enrichment was false discovery rate (FDR) < 0.25 and *p*.adjust < 0.05.

### 2.7. Cell Culture and Real-Time PCR

Human renal cortical proximal tubule epithelial cell HK-2 and human renal carcinoma cell lines 786O, 769P, and Caki-1 were cultured in DMEM/F12, RPMI1640, and McCoy's 5A, respectively, containing 10% fetal bovine serum (FBS) and 1% penicillin-streptomycin. All these cells were incubated in an incubator with 5% CO_2_ at 37°C. Total RNA of cell samples was extracted by TRIzol, after which reverse transcription into cDNA as qPCR template was conducted. The primers APPL1 (forward, 5′-GGACAGCCCGCAGACAAG-3′, reverse, 5′-CCTCCCAATGGAAAACGCTG-3′) and ribosomal RNA 18S (forward, 5′-GTTCCGACCATAAACGATGCC-3′, reverse, 5′-TGGTGGTGCCCTTCCGTCAAT-3′) were used for three independent replicates of qPCR.

### 2.8. Western Blot

For sample preparation, 10^6^ cells were collected and lysed by 120 *μ*L protein loading buffer (2x) and predenatured at 100°C for 10 min. The 12% SDS-PAGE was performed with 4 *μ*L of cell protein samples, and the rest of the steps were performed according to the protocol of Western blot [21, 22]. Antibodies used in the experiment included rabbit monoclonal antibody APPL1 (ab180140, Abcam), mouse monoclonal antibody *α*-tubulin (YM3035, Immunoway), and goat anti-rabbit/mouse HRP (HA1001, HA1006, HUABIO).

### 2.9. Immunohistochemistry (IHC)

Twelve patient samples involved in this study were obtained and used for validation experiment, under the approval by the Ethics Committee of Sichuan Provincial People's Hospital, and informed consent was obtained from the participants. Each tissue sample was fixed in 4% neutral formaldehyde solution for 48 hours, then embedded in paraffin, section, dewaxing, and antigen retrieval, and the rest of steps were completed according to the immunohistochemical protocol [23, 24]. The images were collected by light microscope (200x, Nikon). Antibodies used in the experiment included rabbit monoclonal antibody APPL1 (ab180140, Abcam) and Donkey anti rabbit HRP (bs-0295D, BIOSS). In addition, another IHC images of clear cell carcinoma were obtained from the Human Protein Atlas online database (https://www.proteinatlas.org/learn/dictionary/cell).

### 2.10. Statistical Analysis

The results of UALCAN, TIMER2.0, TISIDB, and Kaplan-Meier plots are displayed with *p* value, HR, and Cox *p* values via a log-rank test. The correlation of APPL1 expression was evaluated by Spearman's or statistical analysis. The heat map of the correlations between APPL1 and its positive-related genes was performed by the R software package with Spearman's correlation. Statistical significance is expressed as ^∗^*p* < 0.05, ^∗∗^*p* < 0.01, and ^∗∗∗^*p* < 0.001.

## 3. Results

### 3.1. APPL1 mRNA Expression Is Downregulated in KIRC Patients

**Figure 1:**
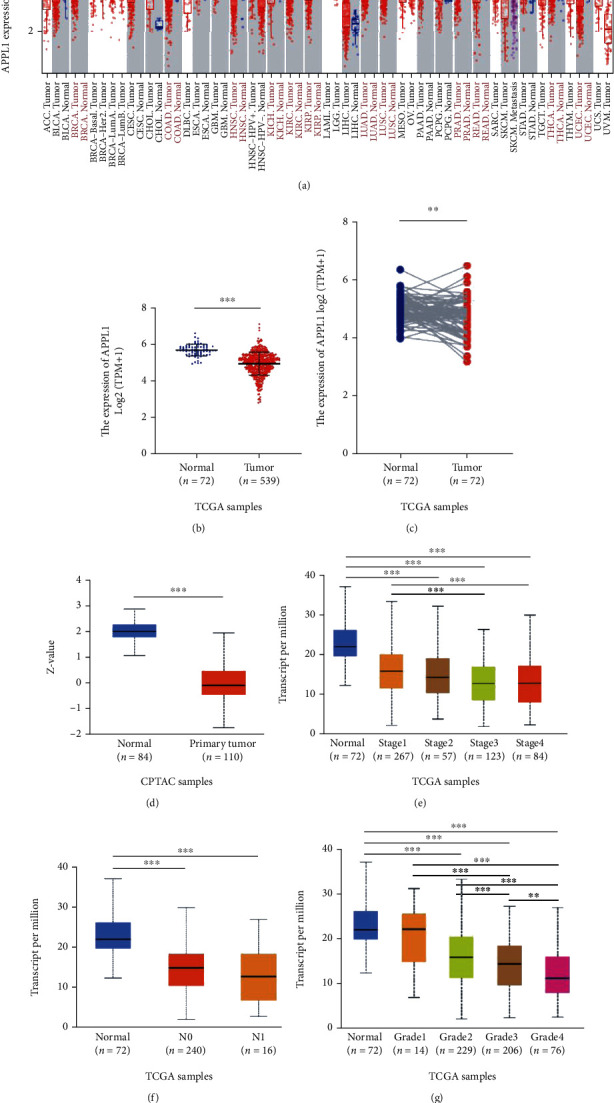
Expression of APPL1 in KIRC. (a) APPL1 expression levels of human different tumor types in the TIMER2.0 database. (b) APPL1 mRNA expression between tumors and normal tissues of KIRC patients from TCGA database. (c) APPL1 mRNA expression between 72 pairs of tumors and adjacent normal tissues of KIRC patients from TCGA database. (d) APPL1 protein expression between primary tumors and normal tissues of KIRC patients from the CPTAC database. (e–g) The downregulation of APPL1 mRNA was significantly correlated with KIRC patients' pathological stage (e), N classification (f), and histological grade (g).

To explore the roles of APPL1 in tumor, we analyzed the expression level of APPL1 mRNA in various human tumor tissues via the Tumor Immune Estimation Resource (TIMER2.0) online database. The results showed that APPL1 mRNA was significantly downregulated in thirteen tumor tissues, which included breast invasive carcinoma (BRCA), colon adenocarcinoma (COAD), esophageal carcinoma (ESCA), head and neck squamous cell carcinoma (HNSC), kidney chromophobe (KICH), kidney renal clear cell carcinoma (KIRC), kidney renal papillary cell carcinoma (KIRP), lung adenocarcinoma (LUAD), lung squamous cell carcinoma (LUSC), prostate adenocarcinoma (PRAD), rectum adenocarcinoma (READ), thyroid carcinoma (THCA), and uterine corpus endometrial carcinoma (UCEC) (^∗^*p* < 0.05 and ^∗∗∗^*p* < 0.001) (Figure 1(a)). We included the following two sets of clinical data from the TCGA database and conducted analysis for KIRC tumor tissues (tumor: 539 cases, normal: 72 cases; clinical information for the samples is included in Supplementary Table 1) and 72 pairs of KIRC tissues (tumor/normal). The results showed that APPL1 mRNA expression of tumor tissues was significantly lower than that of adjacent normal tissues (^∗∗^*p* < 0.01 and ^∗∗∗^*p* < 0.001) (Figures 1(b) and 1(c)). Meanwhile, the trend of protein APPL1 expression in KIRC tumor from CPTAC cohort was consistent with the mRNA level (^∗∗∗^*p* < 0.001) (Figure 1(d)).

To further investigate the variations in APPL1 expression during the progression of KIRC, we analyzed different pathological stages of KIRC patients from the UALCAN database. As expected, the results showed that the expression of APPL1 mRNA was significantly downregulated, with the progression of pathological stage, N classification, and histological grade in KIRC (^∗∗^*p* < 0.01 and ^∗∗∗^*p* < 0.001) (Figures 1(e)–1(g)). These results confirmed the correlation between APPL1 and tumor progression.

**Figure 2:**
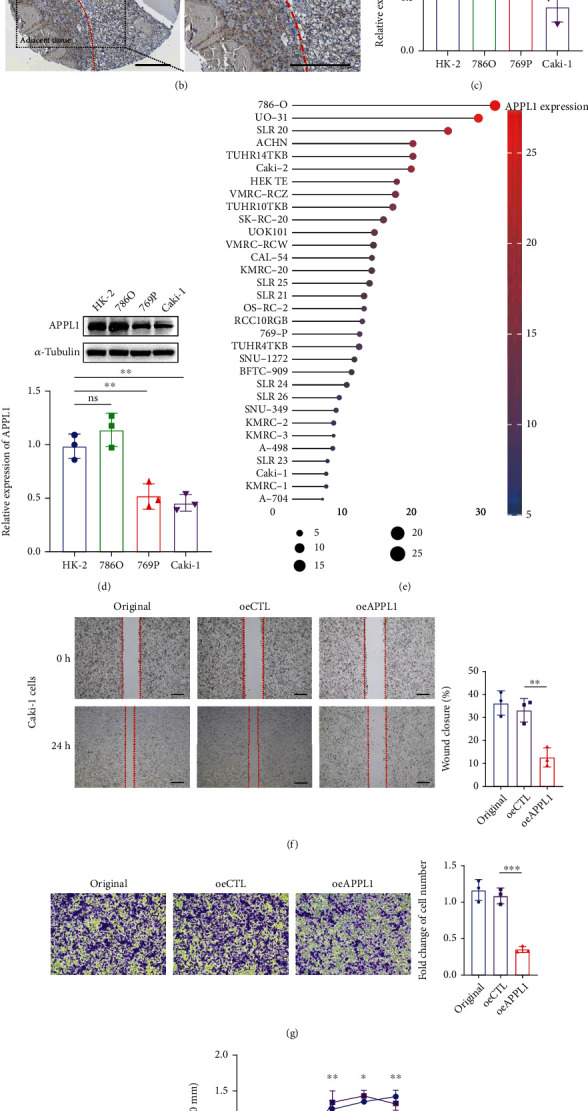
Protein expression of APPL1 in KIRC patients. (a) Immunohistochemical staining of APPL1 in tumor and adjacent tissues form KIRC patients. Dot plots represent statistical quantification with mean ± SD from 24 pairs of KIRC patients (^∗∗∗^*p* < 0.001). (b) Immunohistochemical staining for APPL1 in KIRC sample from the Human Protein Atlas database. (c, d) The mRNA and protein expression levels of APPL1 in HK-2, 768O, 769P, and Caki-1 cell lines were detected by Western blot and qPCR, respectively (^∗^*p* < 0.05 and ^∗∗^*p* < 0.01). (e) Expression levels of APPL1 in different renal cancer cell lines from the CCLE database (different colors or size of dots represent expression level). Scale bar, 100 *μ*m. (f) The scratch wound assay and statistical analysis of caki-1 cells for the group original, oeCTL, and oeAPPL1, respectively (^∗∗^*p* < 0.01). (g) The Transwell assay and statistical analysis of caki-1 cells for the group original, oeCTL, and oeAPPL1, respectively (^∗∗^*p* < 0.01 and ^∗∗∗^*p* < 0.001). (h) The results of cell proliferation assay (CCK8) for the group original, oeCTL, and oeAPPL1, respectively (^∗^*p* < 0.05 and ^∗∗^*p* < 0.01). All experiments were independently repeated three times. This part of the experiment was independently repeated three times.

We further verified the expression of APPL1 in KIRC based on histological and cellular experiments. We found that APPL1 expressed in the cytoplasm was significantly downregulated in tumor tissues relative to normal adjacent tissues (^∗∗∗^*p* < 0.001) (Figures 2(a) and 2(b)). In addition, the expression levels of APPL1 in KIRC tumor cell lines Caki-1 and 769P were significantly lower than that of HK-2 cells (a normal proximal tubular cell line) (^∗^*p* < 0.05 and ^∗∗^*p* < 0.01) (Figures 2(c) and 2(d)), and this result was consistent with the gene expression distribution from CCLE database (Figure 2(e)). The Caki-1 cells belong to the metastatic cell line form KIRC. Based on the difference of APPL1 protein expression between Caki-1 and 786O cells, we speculate that the metastasis of KIRC tumor cells may be related to the expression level of APPL1.

### 3.2. APPL1 Expression Inhibits Caki-1 Cell Migration and Proliferation

APPL1 is expressed at low levels in Caki-1 cells, and we evaluated the effect on metastatic renal cancer cells by restoration to overexpression of APPL1. The results of scratch wound assay showed that the wound closure of the group overexpressing APPL1 (oeAPPL1, 12.81%) was significantly lower than control (oeCTL, 33.18%) for Caki-1 cells (Figure 2(f), ^∗∗^*p* < 0.01). Meanwhile, cell migration assay showed that the cell migration ability of the group oeAPPL1 was significantly lower than oeCTL (^∗∗∗^*p* < 0.001) (Figure 2(g)). Based on these two results, we confirmed that low expression of APPL1 was significantly associated with the migratory ability of Caki-1 cells. In addition, the cell proliferation activity of the group oeAPPL1 was also significantly lower than oeCTL from day 3 to 5 (Figure 2(h), ^∗^*p* < 0.05 and ^∗∗^*p* < 0.01). Altogether, the expression level of APPL1 is associated with the malignant phenotype of KIRC cells and may affect the survival and prognosis of KIRC patients.

### 3.3. Decreased APPL1 Expression Correlates with Poor Prognosis in KIRC Patients

**Figure 3:**
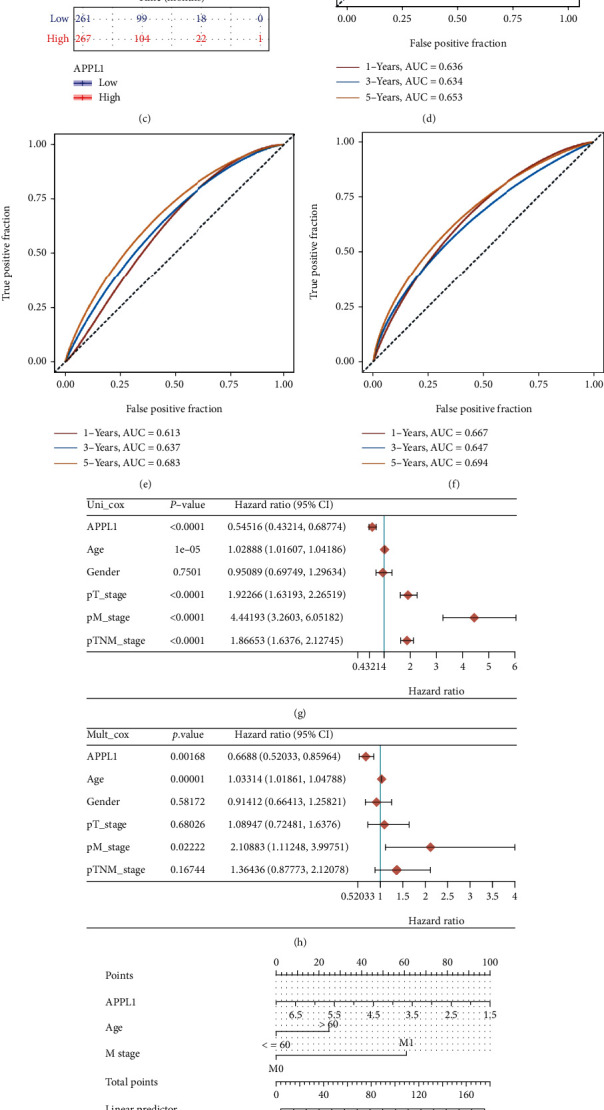
Effect of APPL1 expression on the survival and prognostic value of KIRC patients. (a–c) OS, PFI, and DSS survival curves of KIRC (*n* = 539 and *n* = 72). (d–f) Receiver operating characteristic (ROC) analysis curve of APPL1 for OS, PFI, and DSS survival curves in KIRC. (g) Univariate Cox analysis. (h) Multivariate Cox analysis. (i) Nomogram for OS in KIRC patients. (j) The calibration curves for each year.

Since downregulation of APPL1 was correlated with progression of KIRC, we investigated the effects of APPL1 expression level on the prognosis. Through the Kaplan-Meier plotter (K-M) database, we found that the KIRC patients with low expression of APPL1 had a significantly poor prognosis, which included decreased overall survival (OS), progression-free interval (PFI), and disease specific survival (DSS) (Figures 3(a)–3(c)). To evaluate the accuracy of APPL1 as a prognostic factor, we drew receiver operating characteristic (ROC) curves and calculated area under the curve (AUC) values for APPL1 from TCGA database. As shown in Figures 3(d)–3(f), APPL1, as a potential prognosis factor for KIRC, is of reliability in 1, 3, and 5 years, respectively (OS, AUC > 0.6; PFI, AUC > 0.6; and DSS, AUC > 0.6). Subsequently, the gene APPL1, age, gender, and different classification of KIRC patients were included in univariate and multivariate Cox analyses. The results indicated that APPL1, age, and M stage were significantly independent prognostic factors for OS (Figures 3(g) and 3(h)). Moreover, a nomogram and calibration analysis for KIRC patients also supported the above findings (Figures 3(i) and 3(j)). Taken together, these results suggested that APPL1 may be a prognostic factor for KIRC patients.

### 3.4. Correlation of APPL1 Expression with Treg Cell Infiltration in KIRC

**Figure 4:**
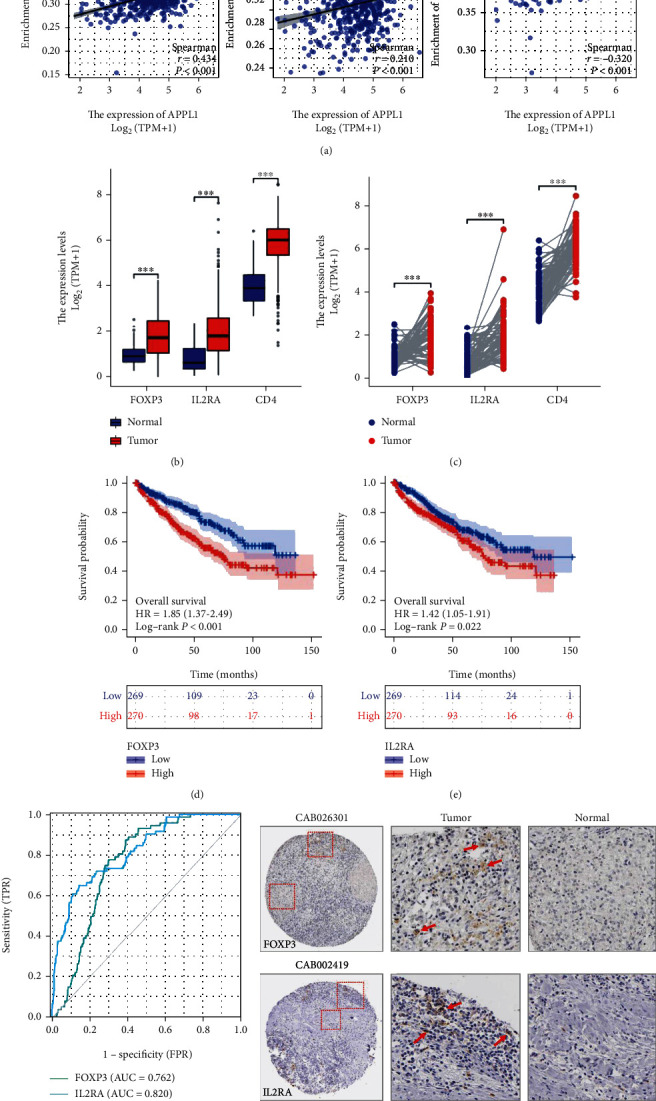
Correlation of APPL1 expression with immune infiltration level in KIRC. (a) The correlation of APPL1 expression with 6 types of T cells. (b, c) The expression difference of Treg cell marker genes FOXP3, IL2RA, and CD4 with unpaired and paired samples in KIRC. (d, e) K-M survival curve of Treg cell marker genes FOXP3 and IL2RA in KIRC. (f) ROC curve of Treg cell marker genes FOXP3 and IL2RA in KIRC. (g, h) Immunohistochemistry of Treg cell marker genes FOXP3 and IL2RA.

KIRC is a heterogeneous tumor with high immune infiltration, which affects tumor microenvironment [25, 26]. Among those immune cells, tumor-infiltrating T lymphocytes are closely correlated with prognostic survival [12]. Therefore, we explored the correlation of between APPL1 expression level and typical T cell infiltration. The results indicated that APPL1 expression level had significantly negative correlation with regulatory T cells (Treg) (*R* = −0.310, *p* < 0.001), CD8^+^ T cells (*R* = −0.193, *p* < 0.001), and total T cells (*R* = −0.104, *p* = 0.016) (Figure 4(a)). Meanwhile, central memory T cells (Tcm) (*R* = 0.434, *p* < 0.001), effector memory T cells (Tem) (*R* = 0.210, *p* < 0.001), and T helper cells (Th) (*R* = 0.320, *p* < 0.001) were positively correlated with APPL1 expression (Figure 4(a)). Treg cells are typical cells of immune infiltration, and the marker genes FOXP3 and IL2RA were significantly upregulated in tumor tissues in paired and unpaired samples (Figures 4(b) and 4(c)), which correlated with poor survival and prognosis of KIRC patients (Figures 4(d)–4(f)). Immunohistochemistry showed the infiltrating distribution of Treg cell marker proteins FOXP3 and IL2RA in renal carcinoma tissue from the Human Protein Atlas database (Figures 4(g) and 4(h)).

KIRC has a higher T cell infiltration score among 19 cancer types detected [25]. To further investigate the mechanism of APPL1 as an important factor in KIRC, T cell populations and APPL1 had been included for correlation analysis via TIMER2.0 database. As expected, the results showed that APPL1 was significantly negatively correlated with exhausted T cells (^∗^*p* < 0.05 and ^∗∗∗^*p* < 0.001) (Table 1). In addition, Th2 and effector T cells were found to have significantly positive correlation with APPL1 expression level (^∗^*p* < 0.05 and ^∗∗∗^*p* < 0.001) (Table 1). In summary, the expression level of APPL1 was negatively correlated with Treg cell infiltration.

### 3.5. Correlation of APPL1 Expression with Immune Checkpoints in KIRC

**Figure 5:**
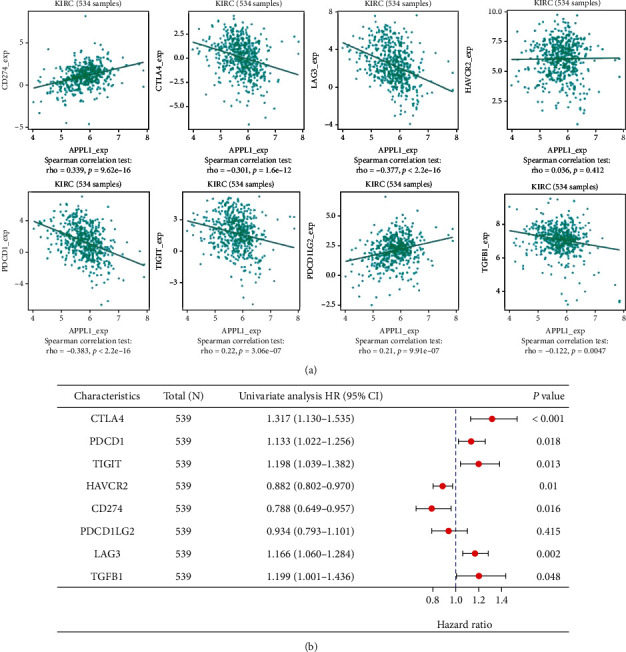
Correlations of APPL1 expression with immune checkpoints and prognostic analysis. (a) Correlations of APPL1 expression with immune checkpoints (CTL4, PDCD1, TIGIT, HAVCR2, CD274, PDCD1LG2, LAG3, and TGFB1). (b) Forest plot shows univariate Cox regression analysis for immune checkpoints in KIRC patients from TCGA database.

Based on the negative correlation between APPL1 expression and Treg cells, we analyzed the correlation between APPL1 expression and eight typical checkpoints for KIRC via TISIDB database. The results showed that the expression of APPL1 was significantly and negatively correlated with immune checkpoints of CTLA4 (*R* = −0.301, *p* < 0.001), LAG3 (*R* = −0.377, *p* < 0.001), PDCD1 (*R* = −0.383, *p* < 0.001), TIGIT (*R* = −0.22, *p* < 0.001), and TGFB1 (*R* = −0.122, *p* < 0.01); was positively correlated with CD274 (*R* = 0.339, *p* < 0.001) and PDCD1LG2 (*R* = 0.21, *p* < 0.001); and had no correlation with HAVCR2 (*R* = 0.036, *p* = 0.412) (Figure 5(a)). These eight immune checkpoints were included in a univariate Cox prognosis regression analysis for KIRC, the results of which showed that others were significantly associated with survival prognosis except for PDCD1LG2 (*p* = 0.415) (Figure 5(b)). These results showed that APPL1 expression was significantly and negatively correlated with immune checkpoints of Treg cells, and these immune checkpoints (CTLA4, LAG3, PDCD1, TIGIT, and TGFB1) may contribute to poor prognosis in KIRC.

### 3.6. Functional Enrichment of Genes Positively Correlated with APPL1 in KIRC

**Figure 6:**
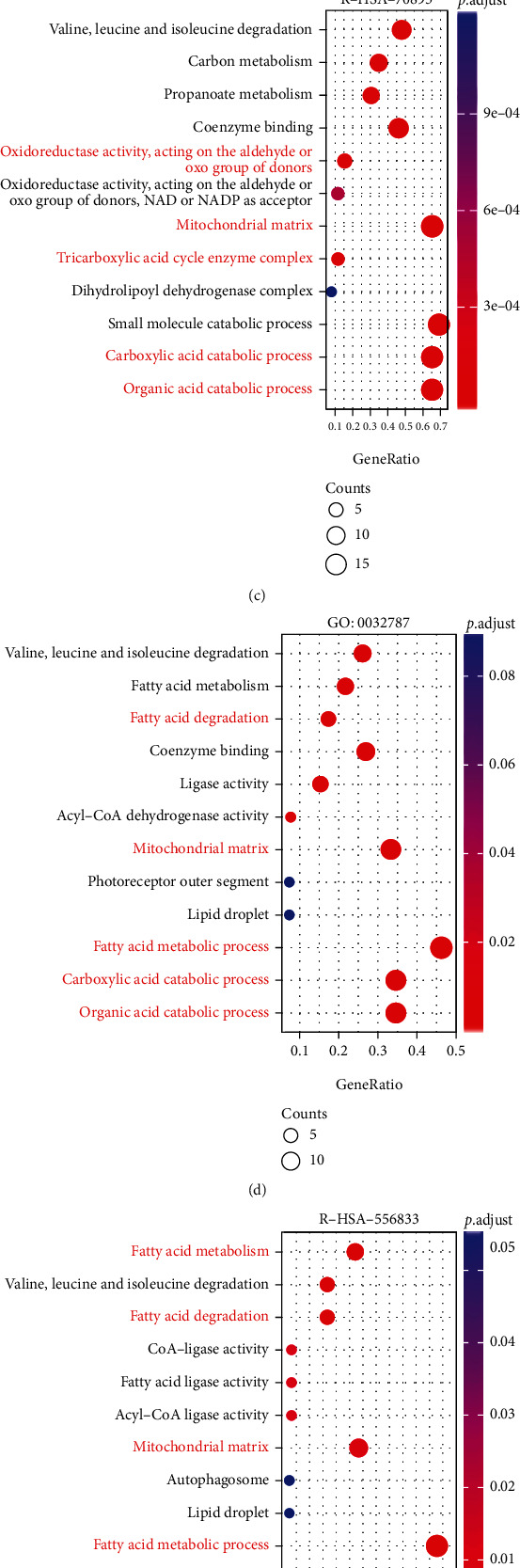
Functional enrichment of APPL1 and its related genes in KIRC. (a) 246 genes shared by between the APPL1-PCGs and downregulation/DEGs in KIRC from UALCAN and TCGA databases, respectively. (b) GO and KEGG enrichment analyses of 246 genes. (c–f) The bubble charts of GO and KEGG enrichment analyses based on pathways of R-HSA-70895, GO: 0032787, R-HSA-556833, and hsa05230, respectively.

To further explore the mechanism of APPL1 for Treg cell infiltration in KIRC, we found 246 genes shared between the 6389 genes that positively correlated with APPL1 (APPL1-PCGs) and 1300 differentially expressed genes (downregulated/differentially expressed genes (DEGs)) in KIRC from UALCAN and TCGA databases, respectively (Figure 6(a)). These genes had been included in GO and KEGG enrichment analyses. The results showed the metabolic pathways of several different substances, which may be involved in energy metabolic processes (Figure 6(b)). We further chose the 4 pathways related to metabolism for energy (R-HSA-70895, GO: 0032787, R-HSA-556833, and hsa05230) to conduct GO and KEGG enrichment analyses, respectively. The results of enrichment focus on some oxygen-consuming metabolic processes, such as carboxylic acid catabolic process, tricarboxylic acid cycle enzyme complex, mitochondrial matrix, fatty acid metabolic process, central carbon metabolism in cancer, cellular respiration, and energy derivation by oxidation of organic compounds (Figures 6(c)–6(f)).

**Figure 7:**
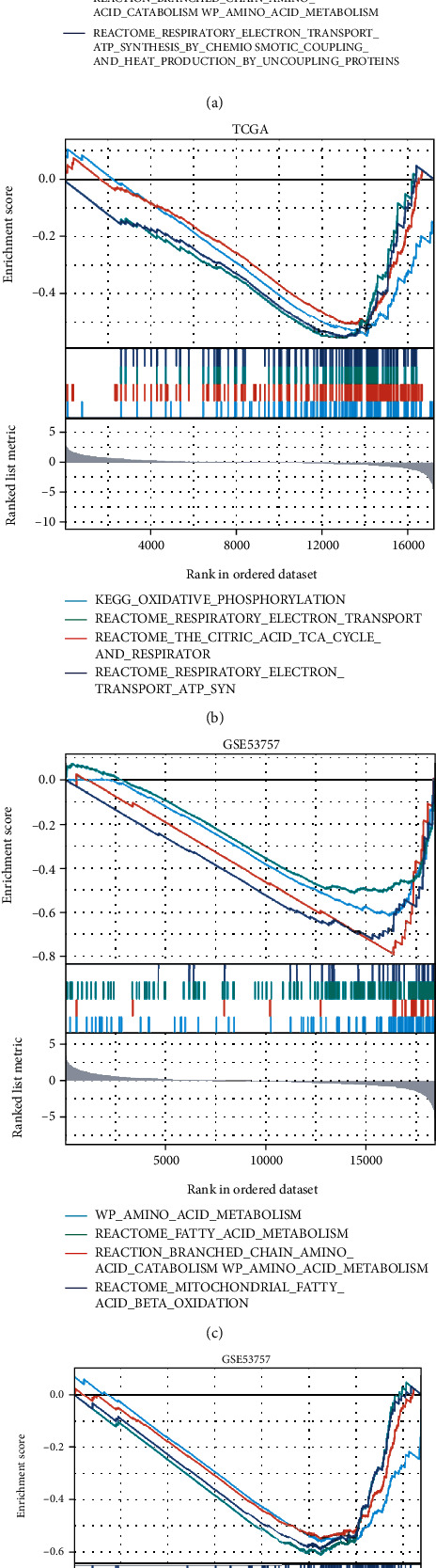
The GSEA enrichment analysis from TCGA and GEO databases for KIRC samples. (a, b) The results of GSEA enrichment for gene set of the amino acid/fatty acid metabolism and oxygen consumption metabolism from TCGA database, respectively. (c, d) The results of GSEA enrichment for gene set of the amino acid/fatty acid metabolism and oxygen consumption metabolism from GSE53757 data, respectively.

In addition, the differentially expressed genes from TCGA and GEO (GSE53757) databases were included in GSEA enrichment analysis, respectively. The results showed that two major processes involved in branched-chain amino acid and fatty acid metabolism were consistent with results of GO/KEGG enrichment, and both showed a significant downregulation trend (FDR < 0.25 and *p*.adjust < 0.05) (Figures 7(a) and 7(c)). Meanwhile, the four processes involved in oxygen-consumption metabolism (KEGG_OXIDATIVE_PHOSPHORYLATION, REACTOME_THE_CITRIC_ACID_TCA_CYCLE_AND_RESPIRATORY_ELECTRON_TRANSPORT, REACTOME_RESPIRATORY_ELECTRON_TRANSPORT, and REACTOME_RESPIRATORY_ELECTRON_TRANSPORT_ATP_SYNTHESIS_BY_CHEMIOSMOTIC_COUPLING_AND_HEAT_PRODUCTION_BY_UNCOUPLING_PROTEINS) were downregulated (FDR < 0.25 and *p*.adjust < 0.05) (Figures 7(b) and 7(d)). Taken together, the low expression of APPL1 and its related genes may be positively correlated with the process of oxygen-consumption metabolism in KIRC.

### 3.7. Correlation between the Oxygen-Consumption Metabolism and Treg/Tcm Infiltration in KIRC

**Figure 8:**
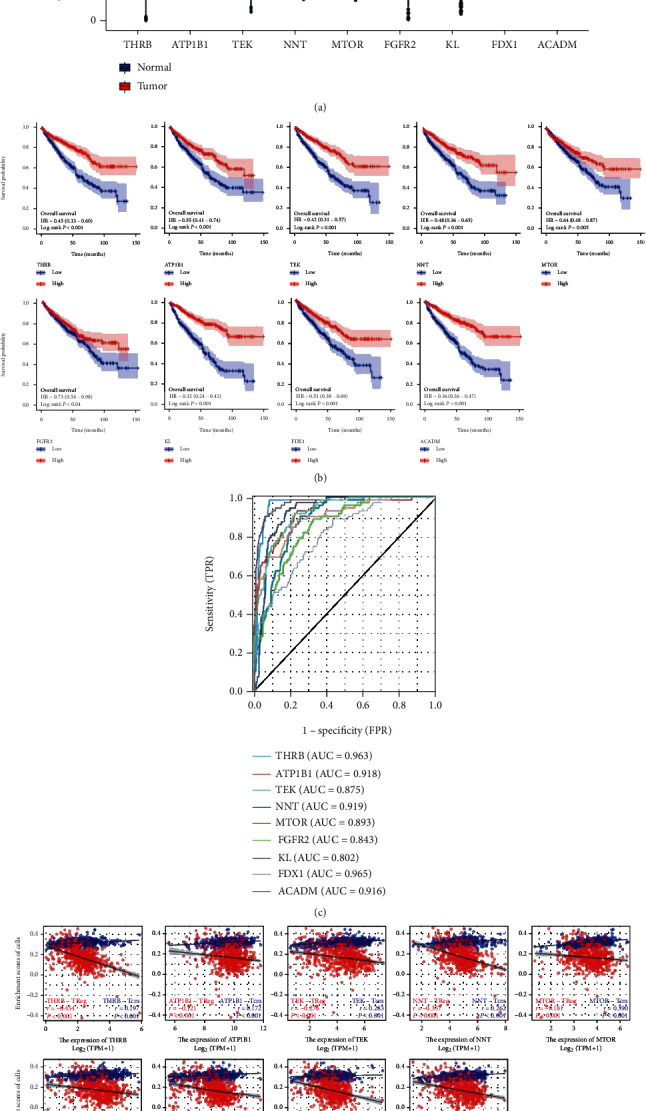
Analysis of survival prognosis and Treg/Tcm cell infiltration from the expression of the 9 genes in KIRC. (a) The expression differences of the 9 genes in KIRC from TCGA database (^∗∗∗^*p* < 0.001). (b) The K-M survival curves of 9 genes, respectively. (c) The ROC curves of the 9 genes, respectively. (d) The correlation analysis of Treg and Tcm cells for the 9 genes, respectively.

To confirm the association between oxygen-consumption metabolism-related genes and Treg/Tcm cell infiltration, we selected the pathway “central carbon metabolism in cancer” as a representative verification and analysis. The central carbon metabolism is the main source of energy required by organisms and plays a major role in oxidative energy supply. Therefore, we screened 9 genes by Cox univariate regression analysis (Supplementary Figure 1A), LASSO regression analysis (Supplementary Figure 1B, C), and analysis of risk factor signature (Supplementary Figure 1D) based on 21 genes from central carbon metabolism in cancer. These genes include THRB, ATP1B1, TEK, NNT, MTOR, FGFR2, KL, FDX1, and ACADM, which showed a significant downregulation trend in KIRC (Figure 8(a)). In addition, there were significant differences in survival and prognosis (Figures 8(b) and 8(c), *p* < 0.05 and AUC > 0.8).

We further assessed the correlation of these 9 genes with immune infiltration used by Treg and Tcm cells, respectively. The results showed that only FDX1 had no significant correlation with Tcm (*R* = 0.002, *p* = 0.969), and others had significantly negative and positive correlations with Tregs and Tcm (*p* < 0.05), respectively (Figure 8(d)). In addition, the immunohistochemical protein levels revealed that 8 proteins were significantly downregulated in renal cancer tissues relative to adjacent tissues from the Human Protein Atlas (THPA) database (Supplementary Figure 2) (the protein KL was not included in the THPA database). Taken together, this part suggested that low expression of 9 genes, which related to oxidative metabolism processes, was associated with immune infiltration and antitumor immunity.

### 3.8. Downregulation of APPL1 Expression Reduces Drug Chemosensitivity via MTOR Pathway

**Figure 9:**
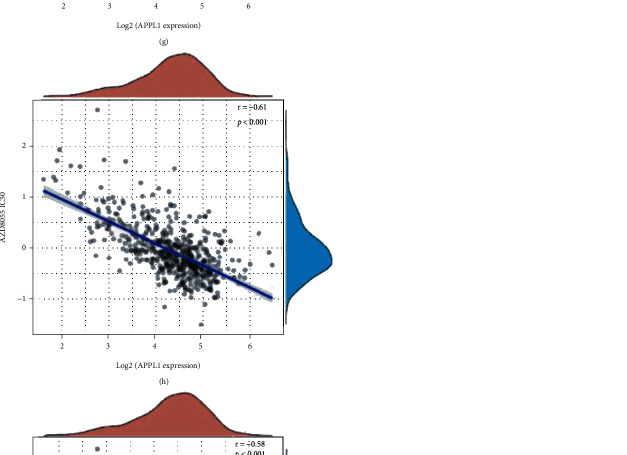
Correlations of APPL1 expression with MTOR pathway and drug chemosensitivity in KIRC. (a–c) Correlations of APPL1 expression with PI3K, AKT1, and MTOR, respectively. (d–i) Correlations of APPL1 expression with drug chemosensitivity of PI-103, PIK-93, AKT inhibitor VIII, MK-2206, AZD8055, and temsirolimus from GDSC database, respectively.

The expression of 9 genes was involved in oxidative metabolism of central carbon in KIRC, including MTOR gene (Figure 8(a)). In addition, MTOR and related pathway nodes may affect the sensitivity of MTOR-targeted chemotherapeutics. Based on the correlation analysis between APPL1 and PI3K/AKT/MTOR in KIRC, we confirmed that APPL1 has a significant positive correlation with the mTOR pathway axis (Figures 9(a)–9(c)). We conduct an online evaluation of the sensitivity of several drugs targeting MTOR based on Genomics of Drug Sensitivity in Cancer (GDSC, https://www.cancerrxgene.org/). The results showed that the sensitivity (IC_50_) of the chemotherapeutic drugs PI-103/PIK-93 (Figures 9(d) and 9(e), *p* < 0.001), AKT inhibitor III/MK-2206 (Figures 9(f) and 9(g), *p* < 0.001), and AZD8055/temsirolimus (Figures 9(f) and 9(g), *p* < 0.001) (targeting PI3K, AKT, and MTOR, respectively) was significantly negatively correlated with the expression level of APPL1. Taken together, downregulation of APPL1 expression in Caki-1 cell line may attenuate the sensitivity of PI3K/AKT/MTOR-targeting drugs during chemotherapy.

## 4. Discussion

Renal cancer is second only to prostate cancer and bladder cancer among the malignant tumors of the urinary system, and about 70% of the kidney cancer are clear cell carcinomas [4, 27]. Immune infiltration and immune evasion are the main causes for poor prognosis in KIRC patients [12]. Therefore, it is important to explore the mechanisms that affect immune infiltration or immunosuppression and find reliable tumor prognostic markers [28]. APPL1 is an adaptor of adiponectin signaling, and it is also closely correlated with the development and differentiation of lymphocytes, such as T cells and macrophages [29, 30]. However, the effects of APPL1 expression on tumor Treg cell infiltration and correlation with poor prognosis have not been reported in KIRC.

In this study, we performed bioinformatics analysis based on the TCGA, UALCAN, TIMER, and TISIDB databases and found that APPL1 was downregulated in KIRC tumor tissues relative to adjacent normal tissues (Figures 1 and 2(a) and 2(b)). Meanwhile, the results of cellular level in vitro also reflected that the expression of APPL1 was associated with the malignant phenotype of tumor cells (Figures 2(f)–2(h)). This finding could also be observed in other 11 types of tumors, which suggesting that downregulation of APPL1 expression may be associated with the development of some tumors. However, this finding was different from a few studies, which found that APPL1 was highly expressed in cholangiocarcinoma (CHOL), liver hepatocellular carcinoma (LIHC), stomach adenocarcinoma (STAD), and breast cancer. Ding et al., Zhai et al., and Liu et al. found that the expression of APPL1 was upregulated in breast cancer (MCF-7) and liver cancer cells (HepG2), and they verified that APPL1 could promote the proliferation and migration of tumor cells via leptin-mediated phosphorylation of STAT3, ERK1/2, and AKT, which could explain the relevant mechanisms [31–33]. The clinical prognosis analysis for KIRC showed that the survival rate was significantly lower in an APPL1-downregulated condition (Figure 3). Thus, these results indicated that APPL1 may be a prognostic factor for KIRC.

Although nephrectomy has always been a common treatment for kidney cancer, 30% of the patients have localization after surgery and then develop into metastases and finally contribute to a high mortality rate [34–36]. Previous studies have shown that APPL1 can inhibit cell migration via inhibiting activation of AKT pathway in tumor [37, 38]. Therefore, downregulation of APPL1 expression may contribute to tumor cell metastasis in KIRC. Moreover, the high aggressiveness of KIRC tumors was mainly associated to the hypoxia induction [39, 40], high infiltration of T cells [41, 42], Th2 cells, and macrophages from tumor microenvironment [25, 43–45]. We found that APPL1 expression was significantly and negatively correlated with Treg cells in KIRC (Figure 4). These correlations suggest that immune response of antitumor may decreased with downregulation of APPL1, and it was consistent with suboptimal chemotherapy outcome or poor prognosis in KIRC [46]. Meanwhile, immunosuppression is closely related to the immune checkpoints of Treg cells in KIRC microenvironment [43, 47], which is a predominant immunosuppressive effect of tumor [25, 47]. However, the results of correlation analysis indicated that downregulation of APPL1 may lead to upregulation of immune checkpoints (CTLA4, LAG3, PDCD1, TIGIT, and TGFB1) in KIRC (Figure 5 and Table 1). The infiltrating T cells could have some typical phenotype that may be closely related to the tumor metabolic microenvironment in KIRC [48–50], but this study has not yet further explored the molecular mechanism of the effect of APPL1 expression on T cell phenotype transformation in tumor microenvironment.

Our study identified the correlation of APPL1 expression with Treg cell infiltration and poor prognosis in KIRC for the first time. We found the expression of APPL1 associated with oxygen-consuming metabolic process in KIRC (Figure 6(b), the red arrow). The proliferation of renal cancer cells undergoes oxidative metabolic reprogramming during adaptation to the hypoxic tumor microenvironment, such as the downregulation processes of hypoxia-induced fatty acid catabolism [51–53]. We confirmed that expression level of APPL1 may affect the Treg cell infiltration (or exhausted T cells) in KIRC via downregulated the processes of oxygen-consuming metabolism in tumor microenvironment. (Figures 6(c)–6(f), 7(c) and 7(d), and 8(d)). However, we still need to further verify this conclusion via restoration experiments. In addition, gene MTOR belong to one of the 9 genes related to oxidative metabolism of central carbon, and the expression level of APPL1 may decrease chemosensitivity of KIRC targeting PI3K/AKT/MTOR (Figure 9). Therefore, low expression of APPL1 may be a reason for chemoresistance in KIRC.

In conclusion, we preliminarily explored the relationship between APPL1 and KIRC and the effect of APPL1 expression. The APPL1 was significantly downregulated with tumor progression in KIRC tissues and might lead to increased infiltration level of Treg cells. Meanwhile, downregulation of APPL1 expression may affect the sensitivity of chemotherapeutics, and it may have contributed to the poor prognosis of KIRC patients. However, we need to further verify or explore the correlation of APPL1 expression level on different subtypes of KIRC and deeper impact mechanism of immune infiltration based on animal level. In summary, APPL1 may serve as a potential biomarker for the diagnosis or prognosis of KIRC and a potential immunotherapy target.

## Figures and Tables

**Table 1:** Correlation analysis between APPL1 and gene markers of different types of T cells form TIMER.

Description	Gene markers	KIRC			
		None		Purity	
		Cor	*p*	Cor	*p*
Th1	TBX21	0.039	0.367	0.047	0.312
	STAT4	0.019	0.662	0.033	0.481
	STAT1	0.338	^∗∗∗^	0.340	^∗∗∗^
	TNF	0.067	0.124	0.078	0.0925

Th2	STAT6	0.397	^∗∗∗^	0.384	^∗∗∗^
	STAT5A	0.227	^∗∗∗^	0.253	^∗∗∗^

Effector T cell	CX3CR1	0.431	^∗∗∗^	0.447	^∗∗∗^
	FGFBP2	0.221	^∗∗∗^	0.224	^∗∗∗^
	FCGR3A	0.235	^∗∗∗^	0.25	^∗∗∗^

Naïve T cell	CCR7	0.022	0.613	0.015	0.755
	SELL	0.222	^∗∗∗^	0.244	^∗∗∗^

Exhausted T cell	HAVCR2	0.201	^∗∗∗^	0.193	^∗∗∗^
	LAG3	-0.214	^∗∗∗^	-0.221	^∗∗∗^
	CXCL13	-0.185	^∗∗∗^	-0.189	^∗∗∗^
	PD-1 (PDCD1)	-0.219	^∗∗∗^	-0.225	^∗∗∗^
	CTLA4	-0.110	^∗^	-0.097	^∗^
	GZMB	-0.148	^∗∗∗^	-0.16	^∗∗∗^

^∗^ *p* < 0.05, ^∗∗^*p* < 0.01, and ^∗∗∗^*p* < 0.001.

## Data Availability

All datasets generated and analyzed during the current study are available from the corresponding authors on request.

## Supplementary Materials

Supplementary Materials Supplementary Figure 1: (A) the univariate Cox analysis of the 21 genes from pathway of GO and KEGG enrichment analyses. (B) LASSO regression coefficients over different values of the penalty parameter for the 21 genes in KIRC. (C) Cross validation plot for the penalty term based on 21 genes in KIRC. (D) The predictive model analysis of risk score for the 21 genes. Supplementary Figure 2: the immunohistochemistry of the 8 gene expressions in renal cancer from THPA database, respectively. Supplementary Table 1: characteristics of patients with KIRC.

## References

[1] Siegel R. L., Miller K. D., Jemal A. (2020). Cancer statistics, 2020. *CA: a Cancer Journal for Clinicians*.

[2] Siegel R. L., Miller K. D., Fuchs H. E., Jemal A. (2021). Cancer statistics, 2021. *CA: a Cancer Journal for Clinicians*.

[3] Bray F., Ferlay J., Soerjomataram I., Siegel R. L., Torre L. A., Jemal A. (2018). Global cancer statistics 2018: GLOBOCAN estimates of incidence and mortality worldwide for 36 cancers in 185 countries. *CA: a Cancer Journal for Clinicians*.

[4] Jonasch E., Walker C. L., Rathmell W. K. (2021). Clear cell renal cell carcinoma ontogeny and mechanisms of lethality. *Nature Reviews. Nephrology*.

[5] Xu W., Atkins M. B., McDermott D. F. (2020). Checkpoint inhibitor immunotherapy in kidney cancer. *Nature Reviews. Urology*.

[6] Gupta K., Miller J. D., Li J. Z., Russell M. W., Charbonneau C. (2008). Epidemiologic and socioeconomic burden of metastatic renal cell carcinoma (mRCC): a literature review. *Cancer Treatment Reviews*.

[7] Song Q., Zheng Y., Wu J. (2021). PTP4A3 is a prognostic biomarker correlated with immune infiltrates in papillary renal cell carcinoma. *Frontiers in Immunology*.

[8] Diamond E., Molina A. M., Carbonaro M. (2015). Cytotoxic chemotherapy in the treatment of advanced renal cell carcinoma in the era of targeted therapy. *Critical Reviews in Oncology/Hematology*.

[9] Zheng J. M., Gan M. F., Yu H. Y. (2021). KDF1, a novel tumor suppressor in clear cell renal cell carcinoma. *Frontiers in Oncology*.

[10] Shuch B., Amin A., Armstrong A. J. (2015). Understanding pathologic variants of renal cell carcinoma: distilling therapeutic opportunities from biologic complexity. *European Urology*.

[11] Wettersten H. I., Aboud O. A., Lara P. N., Weiss R. H. (2017). Metabolic reprogramming in clear cell renal cell carcinoma. *Nature Reviews. Nephrology*.

[12] Diaz-Montero C. M., Rini B. I., Finke J. H. (2020). The immunology of renal cell carcinoma. *Nature Reviews. Nephrology*.

[13] Matsushita H., Vesely M. D., Koboldt D. C. (2012). Cancer exome analysis reveals a T-cell-dependent mechanism of cancer immunoediting. *Nature*.

[14] Zhu Q., Cai M. Y., Weng D. S. (2019). PD-L1 expression patterns in tumour cells and their association with CD8(+) tumour infiltrating lymphocytes in clear cell renal cell carcinoma. *Journal of Cancer*.

[15] Miaczynska M., Christoforidis S., Giner A. (2004). APPL proteins link Rab5 to nuclear signal transduction via an endosomal compartment. *Cell*.

[16] Schenck A., Goto-Silva L., Collinet C. (2008). The endosomal protein Appl1 mediates Akt substrate specificity and cell survival in vertebrate development. *Cell*.

[17] Tan Y., You H., Wu C., Altomare D. A., Testa J. R. (2010). Appl1 is dispensable for mouse development, and loss of Appl1 has growth factor-selective effects on Akt signaling in murine embryonic fibroblasts. *The Journal of Biological Chemistry*.

[18] Sayeed M., Gautam S., Verma D. P. (2018). A collagen domain-derived short adiponectin peptide activates APPL1 and AMPK signaling pathways and improves glucose and fatty acid metabolisms. *The Journal of Biological Chemistry*.

[19] Luan X., Yan Y., Zheng Q. (2020). Excessive reactive oxygen species induce apoptosis via the APPL1-Nrf2/HO-1 antioxidant signalling pathway in trophoblasts with missed abortion. *Life Sciences*.

[20] Zhou Y., Zhou B., Pache L. (2019). Metascape provides a biologist-oriented resource for the analysis of systems-level datasets. *Nature Communications*.

[21] Kurien B. T., Scofield R. H. (2006). Western blotting. *Methods*.

[22] Gallagher S., Chakavarti D. (2008). Immunoblot analysis. *Journal of Visualized Experiments*.

[23] Hofman F. M., Taylor C. R. (2013). Immunohistochemistry. *Current Protocols in Immunology*.

[24] Ella-Tongwiis P., Makanga A., Shergill I., Fôn Hughes S. (2021). Optimisation and validation of immunohistochemistry protocols for cancer research. *Histology and Histopathology*.

[25] Şenbabaoğlu Y., Gejman R. S., Winer A. G. (2016). Tumor immune microenvironment characterization in clear cell renal cell carcinoma identifies prognostic and immunotherapeutically relevant messenger RNA signatures. *Genome Biology*.

[26] Geissler K., Fornara P., Lautenschläger C., Holzhausen H. J., Seliger B., Riemann D. (2015). Immune signature of tumor infiltrating immune cells in renal cancer. *Oncoimmunology*.

[27] DeSantis C. E., Ma J., Gaudet M. M. (2019). Breast cancer statistics, 2019. *CA: a Cancer Journal for Clinicians*.

[28] Kim M. C., Jin Z., Kolb R. (2021). Updates on immunotherapy and immune landscape in renal clear cell carcinoma. *Cancers*.

[29] Tan Y., You H., Coffey F. J., Wiest D. L., Testa J. R. (2010). Appl1 is dispensable for Akt signaling in vivo and mouse T-cell development. *Genesis*.

[30] Chau T. L., Göktuna S. I., Rammal A. (2015). A role for APPL1 in TLR3/4-dependent TBK1 and IKK*ε* activation in macrophages. *Journal of Immunology*.

[31] Ding Y., Cao Y., Wang B. (2016). APPL1-mediating leptin signaling contributes to proliferation and migration of cancer cells. *PLoS One*.

[32] Zhai J. S., Song J. G., Zhu C. H., Wu K., Yao Y., Li N. (2016). Expression of APPL1 is correlated with clinicopathologic characteristics and poor prognosis in patients with gastric cancer. *Current Oncology*.

[33] Liu Y., Zhang C., Zhao L. (2017). APPL1 promotes the migration of gastric cancer cells by regulating Akt2 phosphorylation. *International Journal of Oncology*.

[34] Frank I. G., Blute M. L., Cheville J. C., Lohse C. M., Weaver A. L., Zincke H. (2002). An outcome prediction model for patients with clear cell renal cell carcinoma treated with radical nephrectomy based on tumor stage, size, grade and necrosis: the SSIGN score. *The Journal of Urology*.

[35] Wolff I., May M., Hoschke B. (2016). Do we need new high-risk criteria for surgically treated renal cancer patients to improve the outcome of future clinical trials in the adjuvant setting? Results of a comprehensive analysis based on the multicenter CORONA database. *European Journal of Surgical Oncology*.

[36] Meskawi M., Sun M., Trinh Q. D. (2012). A review of integrated staging systems for renal cell carcinoma. *European Urology*.

[37] Diggins N. L., Webb D. J. (2017). APPL1 is a multifunctional endosomal signaling adaptor protein. *Biochemical Society Transactions*.

[38] Broussard J. A., Lin W. H., Majumdar D. (2012). The endosomal adaptor protein APPL1 impairs the turnover of leading edge adhesions to regulate cell migration. *Molecular Biology of the Cell*.

[39] Iliopoulos O. (2006). Molecular biology of renal cell cancer and the identification of therapeutic targets. *Journal of Clinical Oncology*.

[40] Kaelin W. G. (2008). The von Hippel-Lindau tumour suppressor protein: O_2_ sensing and cancer. *Nature Reviews. Cancer*.

[41] Siska P. J., Beckermann K. E., Mason F. M. (2017). Mitochondrial dysregulation and glycolytic insufficiency functionally impair CD8 T cells infiltrating human renal cell carcinoma. *Insight*.

[42] Ricketts C. J., De Cubas A. A., Fan H. (2018). The cancer genome atlas comprehensive molecular characterization of renal cell carcinoma. *Cell Reports*.

[43] Chevrier S., Levine J. H., Zanotelli V. R. T. (2017). An immune atlas of clear cell renal cell carcinoma. *Cell*.

[44] Kovaleva O. V., Samoilova D. V., Shitova M. S., Gratchev A. (2016). Tumor associated macrophages in kidney cancer. *Analytical Cellular Pathology (Amsterdam)*.

[45] Motoshima T., Miura Y., Wakigami N. (2018). Phenotypical change of tumor-associated macrophages in metastatic lesions of clear cell renal cell carcinoma. *Medical Molecular Morphology*.

[46] McDermott D. F. (2009). Retracted: immunotherapy of metastatic renal cell carcinoma. *Cancer*.

[47] Vuong L., Kotecha R. R., Voss M. H., Hakimi A. A. (2019). Tumor microenvironment dynamics in clear-cell renal cell carcinoma. *Cancer Discovery*.

[48] Sharpe A. H., Pauken K. E. (2018). The diverse functions of the PD1 inhibitory pathway. *Nature Reviews. Immunology*.

[49] Paterson A. M., Lovitch S. B., Sage P. T. (2015). Deletion of CTLA-4 on regulatory T cells during adulthood leads to resistance to autoimmunity. *The Journal of Experimental Medicine*.

[50] Chen S., Crabill G. A., Pritchard T. S. (2019). Mechanisms regulating PD-L1 expression on tumor and immune cells. *Journal for Immunotherapy of Cancer*.

[51] Du W., Zhang L., Brett-Morris A. (2017). HIF drives lipid deposition and cancer in ccRCC via repression of fatty acid metabolism. *Nature Communications*.

[52] Schödel J., Grampp S., Maher E. R. (2016). Hypoxia, hypoxia-inducible transcription factors, and renal cancer. *European Urology*.

[53] Wettersten H. I. (2020). Reprogramming of metabolism in kidney cancer. *Seminars in Nephrology*.

