# Supplementary material for: APPL1 Is a Prognostic Biomarker and Correlated with Treg Cell Infiltration via Oxygen-Consuming Metabolism in Renal Clear Cell Carcinoma
Source: Oxid Med Cell Longev. 2023 Feb 17;2023:5885203. doi: 10.1155/2023/5885203 (PMC9957629; doi:10.1155/2023/5885203)

Supplementary Table 1. Characteristics of patients with KIRC

Characteristics	Variable	Patients (530)	Percentages (%)
**Vital status**	Alive	366	67.90
	Dead	173	32.10
**Age**	Mean (SD)	60.6 (12.1)	49.53
	Median [Min Max]	61 [26 09]	50.47
**Gender**	Female	186	34.51
	Male	353	65.49
**Pathological stage**	I	267	50.28
	II	57	10.73
	III	123	23.16
	IV	84	15.82
**T classification**	T1	271	51.13
	T2	69	13.02
	T3	179	33.77
	T4	11	2.08
**N classification**	N0	240	45.20
	N1	16	3.01
	NX	275	51.79
**M classification**	M0	440	83.02
	M1	80	15.09
	MX	10	1.89
**Tumor grade**	G1	14	2.64
	G2	229	43.21
	G3	206	38.87
	G4	76	14.34
	GX	5	0.94

Supplementary Figure 1

A

Characteristics	Total(N)	Univariate analysis HR(95% CI)	P value
THRB	539	0.571 (0.479-0.680)	<0.001
ATP1B1	539	0.651 (0.556-0.763)	<0.001
TEK	539	0.658 (0.590-0.733)	<0.001
HIF1A	539	0.905 (0.758-1.082)	0.273
MTOR	539	0.709 (0.563-0.892)	0.003
FGFR2	539	0.854 (0.744-0.981)	0.026
NDUFS1	539	0.586 (0.479-0.716)	<0.001
CAT	539	0.665 (0.582-0.761)	<0.001
PFKM	539	0.701 (0.578-0.849)	<0.001
SUCLG2	539	0.596 (0.484-0.733)	<0.001
FECH	539	0.572 (0.467-0.702)	<0.001
TBC1D4	539	0.652 (0.552-0.770)	<0.001
CROT	539	0.550 (0.431-0.702)	<0.001
PRKCA	539	0.939 (0.791-1.116)	0.477
ERBB2	539	0.657 (0.557-0.775)	<0.001
NNT	539	0.613 (0.522-0.720)	<0.001
KL	539	0.761 (0.703-0.825)	<0.001
FDX1	539	0.488 (0.375-0.637)	<0.001
ACADM	539	0.638 (0.561-0.726)	<0.001
PDHB	539	0.553 (0.407-0.751)	<0.001
LDHB	539	0.821 (0.669-1.008)	0.06

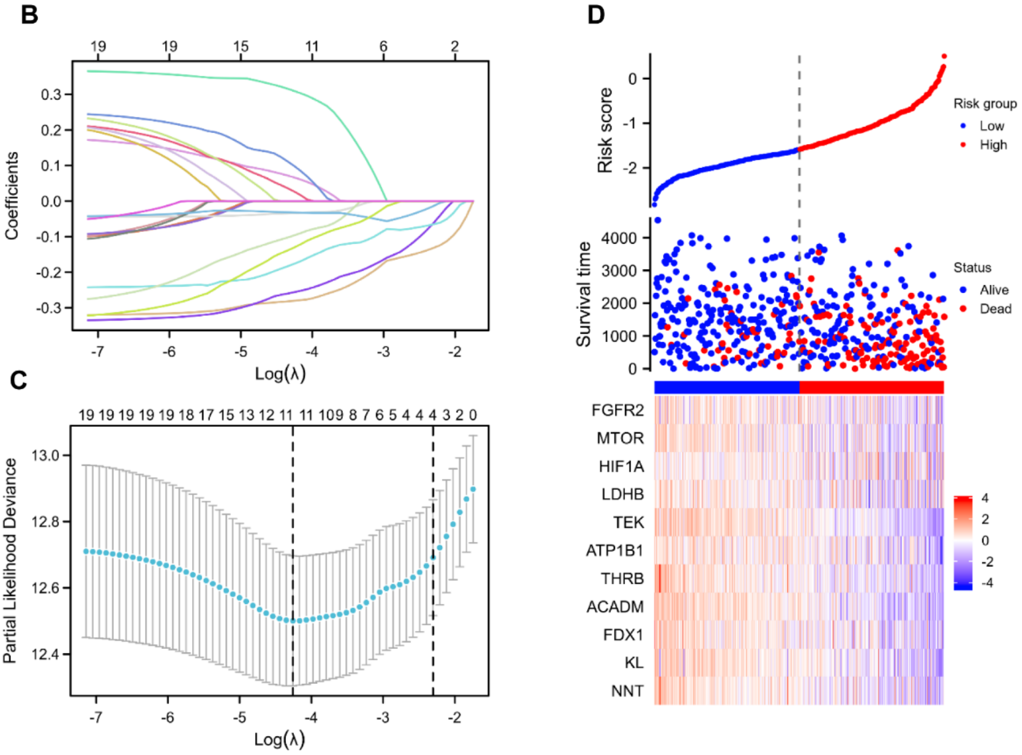

Supplementary Figure 2

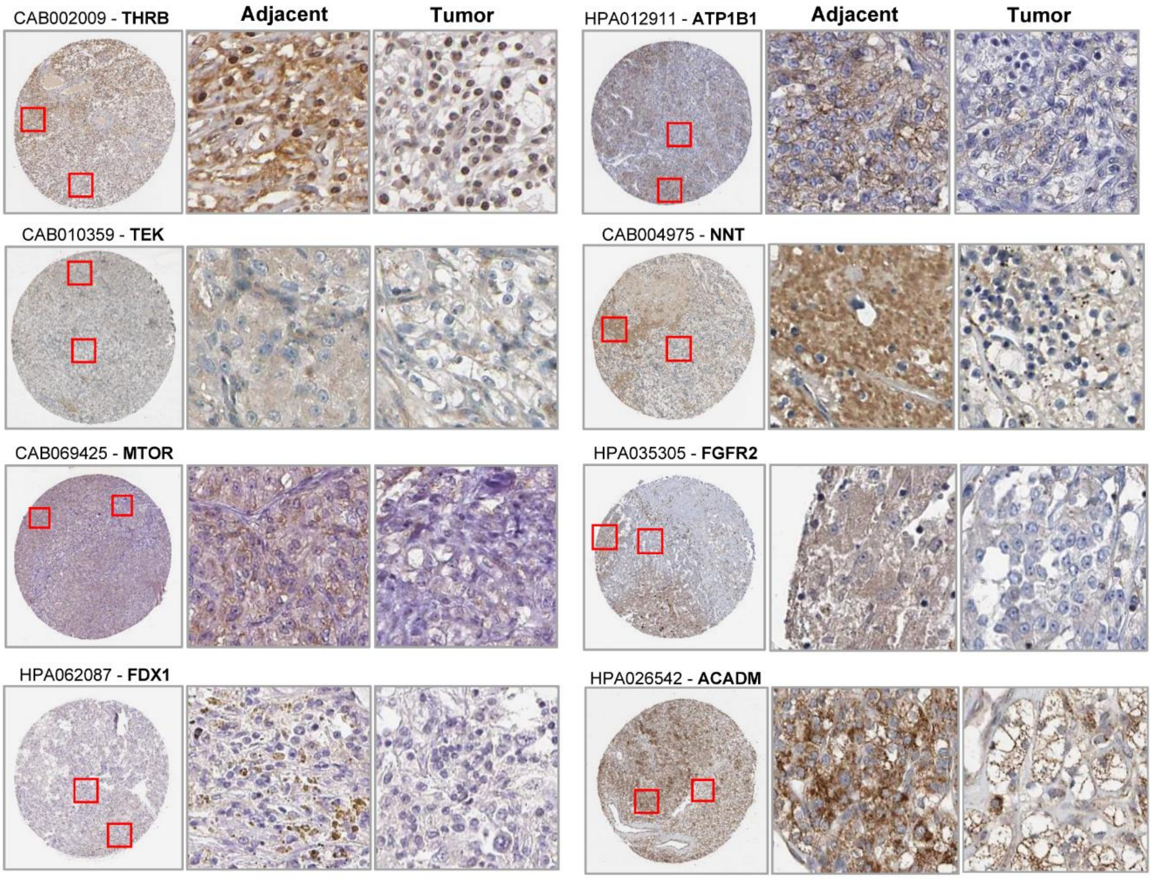

Supplement: Supplementary Materials — Supplementary Figure 1: (A) the univariate Cox analysis of the 21 genes from pathway of GO and KEGG enrichment analyses. (B) LASSO regression coefficients over different values of the penalty parameter for the 21 genes in KIRC. (C) Cross validation plot for the penalty term based on 21 genes in KIRC. (D) The predictive model analysis of risk score for the 21 genes. Supplementary Figure 2: the immunohistochemistry of the 8 gene expressions in renal cancer from THPA database, respectively. Supplementary Table 1: characteristics of patients with KIRC. [file 5885203.f1.pdf]
